# Microbiological Decontamination of Mycotoxins: Opportunities and Limitations

**DOI:** 10.3390/toxins13110819

**Published:** 2021-11-19

**Authors:** Małgorzata Piotrowska

**Affiliations:** Faculty of Biotechnology and Food Sciences, Institute of Fermentation Technology and Microbiology, Lodz University of Technology, Wólczańska 171/173, 90-530 Lodz, Poland; malgorzata.piotrowska@p.lodz.pl

**Keywords:** mycotoxins, decontamination, adsorption, detoxification, microorganisms, lactic acid bacteria, yeasts

## Abstract

The contamination of food and feeds with mycotoxins poses a global health risk to humans and animals, with major economic consequences. Good agricultural and manufacturing practices can help control mycotoxin contamination. Since these actions are not always effective, several methods of decontamination have also been developed, including physical, chemical, and biological methods. Biological decontamination using microorganisms has revealed new opportunities. However, these biological methods require legal regulations and more research before they can be used in food production. Currently, only selected biological methods are acceptable for the decontamination of feed. This review discusses the literature on the use of microorganisms to remove mycotoxins and presents their possible mechanisms of action. Special attention is given to *Saccharomyces cerevisiae* yeast and lactic acid bacteria, and the use of yeast cell wall derivatives.

## 1. Introduction

Mycotoxins are secondary metabolites of fungi that contaminate both plant raw materials and products of plant and animal origin. They can be produced at any stage of the food chain, mainly by fungi belonging to the genera *Aspergillus*, *Penicillium*, *Fusarium*, *Byssochlamys,* or *Alternaria*. Currently, more than 400 compounds are identified as mycotoxins. Most scientific attention has been focused on only a few, due to their frequency and toxic properties. The most important mycotoxins are aflatoxins (AFs), ochratoxin A (OTA), patulin (PAT), citrinin (CIT), *Fusarium* toxins represented by fumonisins (FUM), deoxynivalenol (DON) and their derivatives, zearalenone (ZEN), T-2 and HT-2 toxins (T-2, HT-2), *Alternaria* toxins, such as alternariol (AOH), alternariol methyl ether (AME), tenuazonic acid (TeA), and *Claviceps* ergot alkaloids [1,2].

The main source of human exposure to mycotoxins is food, including not only contaminated food products of plant origin (the primary route), but also contaminated animal tissues (meat, milk, and eggs) derived from animals fed with contaminated fodder. Prolonged exposure to small doses of mycotoxins causes poisoning of various forms, called mycotoxicosis, which may be acute or chronic. Humans and animals are exposed to various types of mycotoxins simultaneously, which may result in antagonistic, additive, or synergistic effects [3]. Mycotoxins have numerous effects on human and animal bodies, causing liver and kidney damage, as well as interfering with the functioning of the digestive tract and the immune system. They may exhibit carcinogenic, mutagenic, cytotoxic, teratogenic, neurotoxic, or estrogenic properties [2]. 

To protect public health in the European Union (EU), the maximum levels of the most toxicologically important mycotoxins permitted in foodstuffs have been established based on the opinions of the European Food Safety Authority (EFSA). EU Regulation 1881/2006 and its amendments (1126/2007, 105/2010, 165/2010, 594/2012 1058/2012, 2015/1137, and 2019/1901) established the maximum acceptable limits for AFs (in nuts, dried fruits, cereals and cereal products, spices, and milk), ochratoxin A (in cereals and cereal products, dried fruits, coffee, grape juice and wine, spices, licorice), patulin (in fruit juices, spirit drinks, cider), fumonisins, deoxynivalenol, zearalenone (in cereals and cereal products, maize and maize-based products, pasta, bread), and citrinin (in food supplements based on rice fermented with *Monascus purpureus*) [4,5,6,7,8,9,10,11]. Similar regulations, though not covering such a wide range of mycotoxins and product categories, are in force in the USA, Canada, Australia, Japan, China, and other countries, as well as in Codex Alimentarius standards. Maximum acceptable limits also apply to feed—e.g., in EU regulations such as Commission Recommendation 2006/576 [12].

Most review publications on the scale of contamination cite Food and Agriculture Organization of the United Nations (FAO) data from 25 years ago that “up to 25% of world food crops are significantly contaminated with mycotoxins” [13]. According to more recent data published by Eskola et al. [14], the number of tested samples that exceed acceptable mycotoxin levels in the European Union (EU) are in line with the FAO estimate.

**Figure 1:**
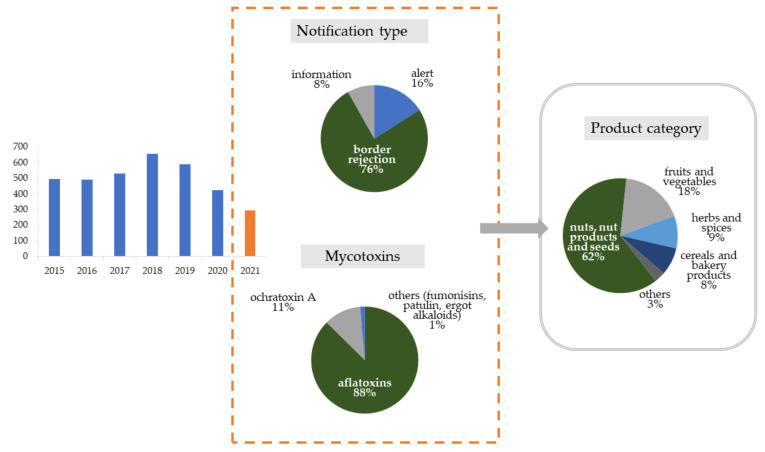
RASFF notifications of excessive levels of mycotoxins from 2015 to 2021. Detailed data on the type of notification, mycotoxins, and products category relate to 2021.

The scale of mycotoxin contamination in foodstuffs analyzed during official food control is reflected in the number of notifications issued by the Rapid Alert System for Food and Feed (RASFF), which is a platform for exchanging information about foods and feeds that may pose a hazard to consumers in Europe. In recent years (since 2015), the RASFF system has registered over 500 notifications of excessive mycotoxin levels in food per year (Figure 1). In 2021, in the period before September, there were 294 notifications, including 16% alert and 8% information notifications. Most notifications were issued by border control (76%). Nuts, nut products, and seeds were the main product categories exposed to contamination (62%).

The scale of the problem appears even greater when we consider levels of mycotoxins in plant materials and food products that are above the limits of detection (LODs) by analytical methods. Not all collected data are published and disseminated by the FAO, World Health Organization (WHO), or EFSA [14]. However, according to Lee and Ryu, 61% of unprocessed food-grade cereals tested worldwide between 2006 and 2016 were contaminated with fumonisins. The incidence of contamination varied from 39% in Europe to 95% in America [15]. The most up-to-date reports on feed contamination are provided by Biomin (Austria). According to their data for the third quarter of 2020 to the second quarter of 2021, the proportions of feed contaminated with mycotoxins were as follows: OTA—9%, T-2—11%, Afs—14%, ZEN—49%, FUM—49%, DON—54%. It should be emphasized that 55% of the feeds were contaminated with more than one mycotoxin, which may increase their toxic effects [16]. The data from Europe are the most complete. However, this information only includes mycotoxins covered by EU regulations—i.e., AFs, OTA, PAT, ZEA, DON, and FUM. The data do not take into account other toxic metabolites, such as *Alternaria* toxins and sterigmatocystin, or masked mycotoxins not detected by routine methods [17].

In addition to its negative impact on human and animal health, mycotoxin contamination has global economic consequences. For this reason, minimizing mycotoxin contamination has become a priority for scientists and organizations including the WHO, FAO, and EFSA. 

## 2. Mycotoxin Control Strategies

**Figure 2:**
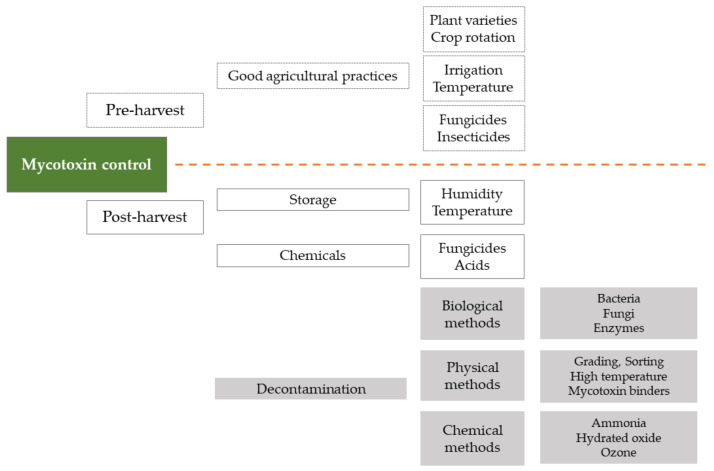
Mycotoxin control strategies.

There are two strategies for the control of mycotoxin contamination (Figure 2): prevention strategies (white boxes) and decontamination strategies (gray boxes). These strategies can be employed at different stages of the production process, often classified as pre-harvest and post-harvest (above and under the dotted line, respectively).

Pre-harvest actions include: the selection of varieties or hybrids resistant to fungal infections and insect pests; minimizing the exposure of plants to stress (drought); careful use of insecticides and herbicides; crop rotation; timely harvest; good soil management to remove, destroy, or bury infected harvest residues. Guidelines for the application of appropriate preventive measures are presented in EU Regulation 1881/2006 and EU Commission Recommendation 583/2006 (on the prevention and reduction of *Fusarium* toxins in cereals and cereal products) [11,18].

Post-harvest methods to prevent the growth of toxicogenic fungi include storage of crops under conditions of appropriate humidity and temperature, or the use of chemical fungicides. If, despite these methods, the products are contaminated with mycotoxins, treatments may be applied to reduce levels of mycotoxins. These include traditional and innovative physical methods (e.g., sorting, thermal treatment, UV radiation, cold plasma, electron beam irradiation, pulsed electric field, adsorbents), as well as chemical methods (addition of oxidants such as hydrogen peroxide, sulfur dioxide, sodium hypochlorite, ozone, or ammonia) [19,20,21].

However, some of these methods are not applicable in practice, mainly due to the risk of creating toxic residues or affecting the nutritional value and organoleptic properties of the purified products [20]. Moreover, there are currently no legal regulations regarding decontamination of food. According to Regulation 1881/2006, foodstuffs that do not comply with accepted maximum levels of toxins should not be used as food ingredients, nor mixed with other foodstuffs, and should not be deliberately detoxified using chemical treatments. The presence of contaminants in food must be reduced as much as possible by Good Manufacturing Practice (GMP), Good Agriculture Practices (GAP), and the application of Hazard Analysis and Critical Points (HACCP). Sorting or other physical treatment methods make it possible to reduce the AF content in groundnuts, nuts, dried fruit, and cereals [11]. The regulations do not mention biological methods of decontamination.

## 3. Background

**Figure 3:**
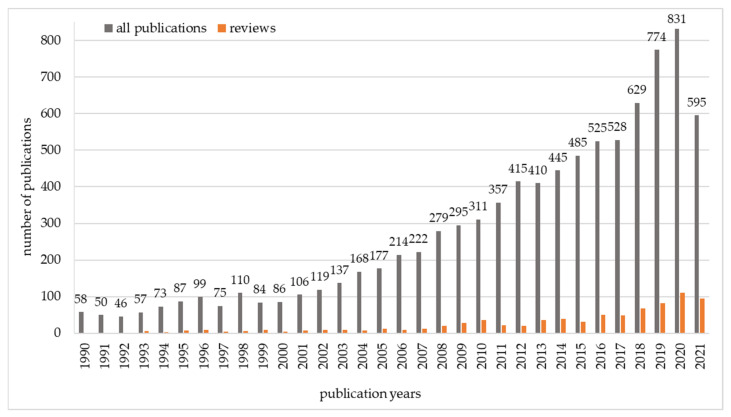
Number of publications on mycotoxin biodegradation from 1990 to 2021, based on the results of searching the Web of Science Core Collection (total = 8854).

The subject of reducing the number of mycotoxins in food and feed is of great interest to scientists. Searching the Web of Science Core Collection for the terms (“mycotoxins” OR “mycotoxin”) AND (“degradation” OR “biodegradation” OR “detoxification” OR “biodetoxification” OR “binding” OR “control” OR “adsorption” OR “elimination” OR “decreasing” OR “minimizing” OR “inactivation” OR “mitigation” OR “transformation” OR “biotransformation” OR “decontamination”) yields 8854 publications from 1990 to the present day. The majority of these publications are original articles. The number of review articles is growing, but they still constitute only 9% of the total number of articles (Figure 3).

A preliminary review of the literature published in the years 1990–2021, using the terms “(bio)degradation”, “(bio)detoxification”, “(bio)transformation”, “decontamination”, “binding”, “control”, “adsorption”, “elimination”, “decreasing”, “minimizing”, “inactivation”, and “mitigation” in combination with “mycotoxins”, identified almost 9000 articles, book chapters, and conference papers in scientific databases (Scopus, Science Direct, Web of Science). The search terms were included in the titles, keywords, and abstracts. Articles published in languages other than English were excluded, as were articles that were unavailable as full texts, and conference papers that had not been peer-reviewed.

In total, 136 scientific papers were selected for discussion in this literature review, of which 66% were published in the years 2010–2021, 28% in 2000–2009, 4% in 1990–2000, and 1% before the 1990s. Thirteen law regulations and one web page were also reviewed. The selected publications were used to answer the following questions: (1) What is the level of research interest in this topic, in terms of the number of publications? (2) What is the current situation, in terms of mycotoxin contamination of food and feed products? (3) What trends can be identified in research on mycotoxin decontamination? (4) Which microorganisms show the greatest potential for use as decontaminants? (5) What are their mechanisms of decontamination? (6) Which methods can be applied in practice?

## 4. Microbiological Methods of Decontamination

The idea of using microorganisms to remove mycotoxins appeared as early as the 1960s. Ciegler et al. [22] reviewed microorganisms in terms of their ability to degrade aflatoxins (AFs). They found that some molds, including *Aspergillus niger*, *Aspergillus parasiticus*, *Aspergillus terreus*, *Aspergillus luchuensis*, *Penicillium reistrickii*, as well as *Flavobacterium aurantiacum* (now *Rhodococcus corynebacterioides*) bacteria are able to transform AFs into a new undefined product. In the following years, it was shown that other microorganisms also exhibit this feature. These include bacteria such as *F. aurantiacum* [23,24,25,26], *Rhodococcus erythropolis*, and *Mycobacterium fluoranthenivorans* [27], *Acinetobacter calcoaceticus* [28], *Bacillus megaterium* [29], *Oenococcus oeni* [30], *Bifidobacterium* sp. [31,32], *Lactobacillus* sp. [31,32,33,34,35,36,37,38,39,40], yeasts such as *Saccharomyces cerevisiae*, *Kluyveromyces marxianus, Rhodotorula rubra, Kloeckera apiculata*, *Candida famata* [41,42,43,44,45,46], and filamentous fungi from *Aspergillus*, *Penicillium*, *Rhizopus*, and *Aureobasidium* genera [47,48,49,50,51].

Given the limitations of physical and chemical methods of decontamination, biological methods using microorganisms or their enzymes are becoming the focus of more research. Approximately 50% of the publications presented in Figure 3 concerned biological methods of decontamination. Until 2010, most publications focused on the search for microorganisms capable of removing aflatoxin B1 (AFB1), and to a lesser extent other mycotoxins (OTA, patulin, and *Fusarium* mycotoxins). The aim was often to reduce the number of mycotoxins under model conditions, in a buffer or microbiological medium, sometimes related to food or feed [30,33,41,48,49]. Some authors described possible decontamination mechanisms, such as enzymatic biotransformation [26,47,50,51] or adsorption to microbial cells [23,34,36,40,41,44,48]. 

After 2010, more advanced research attempted to explain the mechanisms of action by microorganisms. Table 1 presents the subjects of reports from the last 10 years, showing the types of microorganisms, the types of mycotoxins, and the proposed decontamination mechanisms.

As can be seen in Table 1, most studies have focused on the use of bacteria for mycotoxin decontamination. In total, 33 species have been studied from *Alcaligenes*, *Bacillus*, *Brevibacterium*, *Cupriavidus*, *Devosia*, *Escherichia*, *Enterobacter*, *Lysinibacter*, *Lysinibacillus*, *Pediococcus*, *Pseudomonas*, *Rhodococcus,* and *Streptomyces*, as well as lactic acid bacteria. Three consortia of bacteria isolated from soil, compost, and kefir grains were also examined. Fewer studies concerned fungi, most of which focused on the use of yeasts for the decontamination of mycotoxins.

The studies of microbial activity aimed at the removal of mycotoxins discussed so far have been mainly of a scientific nature, allowing for a better understanding of the strains, their properties, and their mechanisms of action, rather than leading to practical applications. Two main methods of microbial decontamination are identified in the literature: adsorption to the cell wall compounds (peptidoglycan, glucomannan, β-D-glucan) and biotransformation to less toxic or non-toxic compounds, thanks to the expression of appropriate enzymes. Biotransformation takes place along different pathways, by the reduction of ketone carbonyl groups, modification of phenolic hydroxyl groups, the hydrolysis of lactone rings, or the creation of connections with glutathione, deamination, or decarboxylation.

The use of microorganisms or their cell components for the decontamination of foods and feeds could have great potential. However, there are still no legal regulations concerning the decontamination of food detoxification processes that can be applied to products destined for use as animal feed. According to Commission Regulation (EC) 386/2009 in the feed technological additives category, a new functional group was created, composed of “substances for reduction of the contamination of feed by mycotoxins”. These are substances that can suppress or reduce the absorption of mycotoxins, promote the excretion of mycotoxins, or modify their mode of action, and thereby mitigate possible adverse effects of mycotoxins on animal health [121]. 

**Figure 4:**
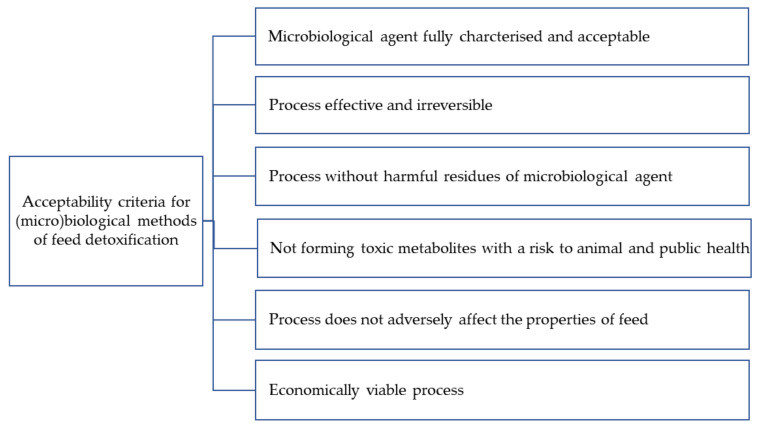
Criteria for acceptability of microbiological decontamination methods.

The rules on the detoxification of feed are contained in Commission Regulation (EU) 2015/786. Acceptable detoxification processes should ensure that feed subjected to detoxification processes does not adversely affect the health of either the farm animals or the consumers of food of animal origin (Figure 4). It should also be effective and irreversible, without changing the properties (e.g., nutritional properties) of the feed. The detoxification process should be performed in a facility approved for the purpose by a competent authority. Only detoxification methods that have obtained a positive EFSA scientific opinion and have been approved by competent institutions may be used [122].

One of the methods of feed detoxification approved by the relevant institutions is the commercial enzyme-based additive FUMzyme^®^, produced by Biomin GmbH, Austria. This product contains fumonisin esterase, produced by a genetically modified *Komagataella pastoris* yeasts strain. The additive is already authorized for use with all pigs, all poultry, and all avian species [123]. According to Rychen et al. [95], when added to feed contamination by FB1, fumonisin esterase is able to significantly reduce the concentration of fumonisin B1 in animal feces and at various points in the digestive tract. This is the result of complete or partial fumonisin de-esterification to less toxic products. FUMzyme^®^ does not have any adverse effect on animal health at the recommended maximum dose 300 U/kg of complete feedstuff. Moreover, it is safe for consumers of animal products [123].

As shown in Figure 4, the first criterion of acceptability for a microbiological decontamination method is a well-characterized and accepted microorganism. Microorganisms that can effectively remove mycotoxins (Table 1) include newly isolated species that have so far been poorly characterized. *Cupriavidus* spp. belonging to the *Burkholderiacea* family are relatively poorly understood bacteria, which can be isolated from soil, root nodules, sewage, and aquatic environments [124]. Other examples of newly isolated microorganisms are *Pelagibacterium halotolerans*, a novel marine halotolerant species of bacteria [125], and *Devosia insulae* [126]. Only microorganisms that are well known, safe, and characterized in terms of pathogenicity can be used for decontamination. Some of the bacteria and yeasts listed in Table 1 can cause infections in humans. These include *Alcaligenes faecalis*, which is often associated with local and systemic infections in humans (endocarditis, bacteremia, meningitis, endophthalmitis, skin and soft tissue infections, urinary tract infections, otitis media, peritonitis, and pneumonia) [127] and *Enterobacter cloceae* complex strains [128,129], as well as *Candida quilliermondii* and *C. parapsilosis*, which are in the group of six pathogenic species of yeast responsible for invasive candidiasis [130]. Most infections caused by the bacteria and yeasts listed in Table 1 are opportunistic infections.

Safe and practical methods that could potentially be acceptable to consumers include the use of lactic acid bacteria and selected species of yeasts or microorganism enzymes. These methods can be used during biotechnological processes for the production of fermented food, such as dairy products, vegetable silages, wine, beer, or sourdough. However, the levels of mycotoxin contamination in the raw materials should still not exceed the accepted levels established in EU Regulation 1881/2006 [11].

### 4.1. Lactic Acid Bacteria

Lactic acid bacteria, including probiotic strains, are of particular interest due to their beneficial physiological effects on human and animal health and their ability bind mutagens from food and the environment [131]. LAB have traditionally been used as natural food and feed preservatives.

Aflatoxin B1, zearalenone, and ochratoxin A have been found to be effectively bound by *Lacticaseibacillus rhamnosus* probiotic strains [31,38,132]. El-Nezami et al. [34,133] showed that this process can be very fast. After only 4 h of contact between the bacteria and AFB1, the initial amount of AFB1 (5 µg/mL) decreased by between 50% and 77%, depending on the strain, pH, temperature, and biomass density. According to these authors, AFB1 was predominantly bound to the carbohydrate components of cells. Hydrophobic and electrostatic interactions played a major role in this process. *Lactobacillus acidophilus* and *L. rhamnosus* strains are also characterized by the ability to remove AFM1 from milk, with effectiveness ranging from 18% to 57%, depending on the strain [33].

The adsorption of ochratoxin A to the cell wall of *Lactiplantibacillus plantarum*, *Levilactobacillus brevis*, and *Fructilactobacillus sanfranciscensis* has been demonstrated in [70]. Using heat-inactivated lactic acid bacteria biomass, the reduction in the amount of the toxin was several times more efficient than using the same density of viable cell biomass. These findings confirm that toxins are adsorbed into the bacterial cells, especially into the peptidoglycan, as in [34]. The better adsorption of mycotoxins by dead cells compared with live cells may be due to changes in the structures of the bacterial cell walls under the influence of high temperature—i.e., denaturation of proteins, generation of pores in the cell wall structure (which increases the permeability of the outer layers of the cell wall), and increased numbers of active areas responsible for the adsorption of various compounds [33,70].

Niderkorn et al. [134] selected lactic and propionic fermentation bacteria for the removal of *Fusarium* toxins from solutions. *Lacticaseibacillus rhamnosus* removed 55% of deoxynivalenol. *Leuconostoc mesenteroides* removed 82% of fumonisin B1, whereas *Lactococcus lactis* removed 100% of fumonisin B1. In vivo experiments showed that the use of a synbiotic preparation with selected probiotic strains of the *Lactobacillaceae* family as feed additives reduced the effects of ochratoxicosis in chickens, as well as having a beneficial influence on the gastrointestinal tract of chickens [135].

Most reports on decontamination by lactic acid bacteria concern aflatoxin B1 and ochratoxin A. The main mechanism responsible for the detoxification of these bacteria is adsorption to the bacterial cell wall. Biotransformations into other products have been reported for patulin and zearalenone (Table 1). Wei et al. [71] tested *Lactiplantibacillus plantarum* strains isolated from traditional Chinese fermented food for their ability to detoxify patulin. One strain, 13M5, showed the ability to transform patulin into less toxic E-ascaladiol. A similar result was obtained by Zheng et al. [72], who used the *Lacticaseibacillus casei* YZU01 strain to remove patulin from apple and pear juice. However, in this case, as well as the main mechanism of biotransformation into E-ascaladiol, adsorption of the toxin into the bacterial cells was observed. In a study by Chen et al. [74], *Lactiplantibacillus plantarum* strains isolated from faeces and the digestive tracts of leaf-nosed bats and ducks were able to degrade ZEA, due to bacterial esterase activity.

The second condition that a microbiological method must meet is the irreversibility of the process (Figure 4). To avoid desorption and re-exposure to toxins, the mycotoxin-adsorbent complex should be stable, especially under gastrointestinal conditions. However, in some cases the adsorbed mycotoxins are released [40,70]. It has been shown in model studies that toxins bound using thermally inactivated LAB cells are more stable than toxins bound using live bacteria [136].

### 4.2. Yeasts

Yeasts are the second group of organisms with important potential applications, especially *Saccharomyces cerevisiae* strains. These organisms are widely used in many biotechnological processes, such as baking, brewing, winemaking, and distilling. Several studies have shown that yeasts can effectively remove different mycotoxins from plant-derived raw materials during fermentation, and in model conditions from microbiological media [41,42,43,44,45,46,106,108,109,110]. 

Patulin in apple and fruit-based food and drink poses a risk to consumer health. Therefore, methods are sought to minimize patulin contamination. Zhang et al. [105] studied patulin adsorption by *Saccharomyces cerevisiae* during fermentation in a model medium spiked with PAT. After 48 h of fermentation, almost 90% of the initial content of PAT was removed. The efficiency of adsorption was found to depend on the duration and temperature of fermentation, as well as the initial PAT concentration. The authors concluded that the toxin was absorbed into the cell wall proteins and polysaccharides. In several studies on patulin removal, a different mechanism was demonstrated. Marine yeast identified as *Kodameae ohmeri* was able to transform PAT to E- and Z-ascladiol. The efficiency of the process was highest at pH 3–6, temperature 35 °C, and an inoculum density of around 5 × 10^8^ cells/mL [94]. After incubation of PAT with *Rhodotorula kratochvilovae*, which is less toxic than PAT, desoxypatulinic acid was formed. The authors suggest that the lower toxicity of desoxypatulinic acid is a consequence of the hydrolysis of the lactone ring and the loss of functional groups that react with thiol groups [101]. In a study by Reddy et al. [97], patulin was effectively degraded by 87.4% after 48 h of fermentation by *Metschnikowia pulcherrima*. Patulin was not detected in the yeast cell walls, which indicates that the yeast did not adsorb PAT but degraded it to an unidentified product of unknown toxicity.

Mycotoxins, especially those produced by *Fusarium* pathogens, pose a problem in breweries. Barley malt can be contaminated with ZEN, DON, and their derivatives FUM and OTA, which can be transferred to malting and brewing by-products [137]. The use of appropriate strains of decontaminable yeast in the production process can improve the quality of the finished products. Nathanail et al. [109] demonstrated that *Saccharomyces pastorianus* lager yeast was able to reduce mycotoxin levels during fermentation of wort naturally contaminated by *Fusarium* trichothecenes. After the 96 h of fermentation, reductions in the numbers of mycotoxins were observed of up to 15% for DON, 17% for DON-3 glucoside, 34% for HT2, and 31% for T2. Since trichothecene metabolites were detected in the beer, the authors suggest that the reactions behind the reduction in mycotoxins may be glucose–sulfate conjugation and deacetylation. Another proposed mechanism was physical binding of the mycotoxins to the yeast cell. Since spent yeast is often used as animal feed, it is important to investigate the stability of the mycotoxin–cell wall complex under gastrointestinal conditions. According to Wall-Martínez et al. [110], the main mechanism of mycotoxin removal during fermentation of contaminated wort is adsorption to the yeast cell wall. After fermentation by the brewer’s yeasts *S. cerevisiae* and *S. pastorianus*, 10–17% of DON and 30–70% of ZEN was removed. The initial concentrations of DON and ZEN in the yeast biomass were 6.4% and 31.3%, respectively. In unfiltered beers, this can be a problem due to the secondary exposure of consumers to mycotoxins, especially as adsorption is reversible at the low pH conditions in the gastrointestinal tract.

Cereals and their derivatives, such as flour and bread, are often contaminated by mycotoxins, mainly OTA and *Fusarium* toxins. The production of sourdough using *Saccharomyces cerevisiae* yeast and lactic acid bacteria reduces the mycotoxin content [38]. Mozaffary et al. [108] found that during dough fermentation *S. cerevisiae* baker’s yeast was able to reduce the amount of OTA in wheat flour by about 60%.

After cereals, the second major source of exposure to OTA is wine. Ochratoxin A contamination is caused by toxigenic fungi such as *Aspergillus carbonarius*, *A. niger*, and *A. awamorii*, which grow on grapes [138]. Certain oenological strains of *Saccharomyces* sp. yeasts are able to remove OTA from grape musts during winemaking [41,106,139,140]. Cecchini et al. [140] demonstrated that, dependending on the yeast strain, wine yeasts are able to remove 46.8–52.2% of the OTA in white wine and 53.2–70.1% of the OTA in red wine during the fermentation process. The absence of degradation products suggested an adsorption mechanism.

The process of removing OTA from grape juice is very fast. In one study, after just 5 min of contact with yeast cells, 90% of the initial toxin content (10 µg/mL) was adsorbed [41]. Similar results were obtained during fermentation of white grape and blackcurrant musts. Fermentation with *Saccharomyces bayanus* resuted in the removal of more than 80% of the initial content of OTA. Heat-inactivated yeast biomass (5 g dry weight/L) adsorbed more than 60% of OTA from wine [106]. Different results, indicating biotransformation, were obtained by Freire et al. [141] during fermentation of grape must artificially contaminated with toxigenic strains of *A. carbonarius* and *A. niger*. The reductions in OTA concentrations ranged from 88.2% to 92.4%, depending on the type of wine. Metabolites such as ochratoxin B, ochratoxin α methyl ester, ochratoxin B methyl ester, ochratoxin A methyl ester, ethylamide ochratoxin A, ochratoxin C, and ochratoxin A glucose ester were also detected. When red grape must contaminated with OTA was fermented by *Metschnikowia pulcherrima*, products of OTA biodegradation (α-OTA and the sodium adduct of α-OTA without the coumarin group) were identified [99].

Some studies have investigated the possibility of using dead yeast cells from appropriate strains as adsorbents in oenological practice [41,106]. Such adsorbents are inexpensive, safe, and do not affect the organoleptic properties of the wine. However, the disposal of the residue is controversial since the toxin can desorb from the yeast cell–OTA complex. Another disadvantage of this method of decontamination is that it binds other ingredients that contribute to wine quality, such as polyphenols and anthocyanins [142]. Petruzzi et al. [143] demonstrated that the process of OTA binding by *Saccharomyces cerevisiae* is reversible and that the stability of the OTA–yeast cell complex depends on the kind of strain, the pH, and the sugar concentration.

## 5. Mycotoxin Adsorbents of Microbial Origin

Another approach to mycotoxin decontamination is the addition of inert dietary supplements to feed, such as clays, kaolin, zeolites, activated carbon, sodium, and magnesium aluminum silicates, as well as hydrated sodium calcium aluminum silicate (HSCAS) or bentonite [144]. These supplements effectively adsorb toxins in the feed or in the digestive tract of animals. As a result, the toxins are not absorbed into the bloodstream and their resorption is prevented. Various inorganic adsorbents are commercially available and some of them are enriched with enzymes. However, the major disadvantage of adsorbents is that they can also bind vitamins, micro- and macro-elements, as well as other essential compounds, thereby reducing the nutritional value of the feed.

The dominant mechanism responsible for the removal of mycotoxins using microorganisms is adsorption to bacterial and yeast cells. Given the many limitations regarding the use of live microorganisms to remove mycotoxins, the use of microbial adsorbents for this purpose offers a promising solution. Most research has focused on preparations containing β-D-glucans extracted from *S. cerevisiae* yeast cell walls. Yeasts cell wall components have been used to adsorb a variety of toxins, including *Fusarium* and *Alternaria* toxins, as well as OTA and AFB1 [42,93,107,111,145,146]. In a study by Bzducha et al. [93], the cell walls and β-glucans isolated from *Candida utilis* were characterized by the greatest ability to bind non-polar mycotoxins, such as ZEN, OTA, and AFB1, especially under acidic conditions. Freimund et al. [42] showed that crosslinked 1,3-β-D-glucan modified by carboxymethyl ether and hexadecyltrimethylammonium salt was able to efficiently adsorb zearalenone and T-2 toxin.

Research on OTA adsorption under model conditions has shown that the polysaccharide fraction of the brewery yeast cell, water-extracted glucan, and commercial glucan adsorbed the highest amounts of OTA, at more than 55% of the initial concentration. Adsorption is most effective at a close-to-neutral pH and is considerably less effective under alkaline conditions. The polysaccharide fraction of the yeast cell wall, namely β-glucans, is responsible for the adsorption of ochratoxin A [107]. Yiannikouris et al. [147] found that zearalenone adsorption provided by β-(1,3)-D-glucans is most effective under acidic and neutral conditions. These conditions are present in some parts of the digestive tract of animals, which suggests that β-(1,3)-D-glucans may be effective as feed additives. Different results were obtained in a study on the adsorption of *Alternaria* toxins (AOH and AME) by thermally deactivated yeasts. In an alkaline environment at pH 9, the toxins were almost completely removed by the yeast powder at a concentration of 40 g/L [111].

Yeasts and their cell wall components are used both as feed additives for animals and as adsorbents that effectively limit mycotoxicosis in farm animals. Raju and Devegowda [148] suggest that the esterified form of β-D-glucan from yeast cell walls can help to protect broiler chickens exposed to aflatoxin B1, ochratoxin A, and T-2 toxin, individually and in combination. The potential application of glucans and yeast cell wall derivatives as mycotoxin adsorbents in feed depends on the stability of the toxin–cell wall complex under the conditions of the gastrointestinal tract. Analysis of the adsorption of OTA by yeast cell wall extract during simulated consecutive digestion steps revealed that more than 80% of the OTA was bound at pH 2.5. The resulting complex was stable after the action of digestive enzymes (pepsin, pancreatin). However, some of the OTA was released when the pH was raised to 6.5. 

An in vivo study on broiler chickens showed that OTA deposits in the livers of chickens given contaminated feed containing cell wall extract were 30% lower after 14 days than the levels of OTA in the control group given contaminated feed without the extract [149]. This result was supported by Ejiofor et al. [150], who found that the addition of 2 g of *S. cerevisiae* yeast to 1 kg of feed neutralized the negative impact of feed naturally contaminated with AFs and DON on the histopathological, hematological, and serum biochemical parameters of chickens, although to a lesser extent than kaolin adsorbent.

Overall, the research literature suggests that adsorbents can be used as functional feed additives, increasing the efficiency and health of poultry exposed to mycotoxins in feed. These findings may be of interest and use to feed producers and livestock breeders. 

## 6. Conclusions

**Figure 5:**
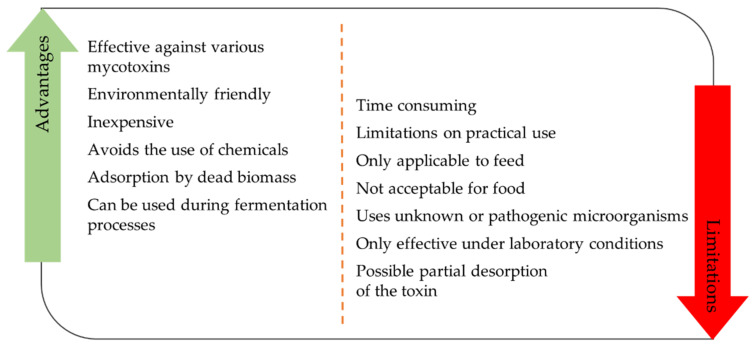
Advantages and limitations of biological methods of decontamination.

This review has surveyed the literature regarding the removal of mycotoxins from food and feed, with a special focus on microbiological methods. Although the decontamination of food using microorganisms has many advantages, there are still no legal regulations concerning the decontamination of food (Figure 5). Biological detoxification processes can, however, be applied to products used as animal feed.

Methods that could be used safely and be acceptable to consumers include the use of lactic acid bacteria and selected yeast species, which can be used in the production of fermented foods such as dairy products, vegetable silages, wine, beer, and sourdough. Selected strains with appropriate technological features could also reduce the content of toxins, increasing the safety of the final product. Another possibility is the addition of dietary supplements to feed, which can effectively adsorb toxins directly in the feed or in the digestive tract of animals. As a result, the toxins are not absorbed into the bloodstream. Yeasts and their cell wall derivatives can be used to adsorb a variety of toxins, including *Fusarium* and *Alternaria* toxins, as well as OTA and AFB1. More research is needed to ensure that these methods meet the many standards required for practical usage. The environmental risk of residues containing toxins, as well as economic aspects, should also be considered.

## Figures and Tables

**Table 1:** Reports concerning the microbial decontamination of mycotoxins in the years 2011–2021.

Microorganisms	Targeted Mycotoxins	Mechanism	References
Bacteria			
*Alcaligenes faecalis*	OTA	Biodegradation to OTα	[52]
*Bacillus amyloliquefaciens*	OTA	Biodegradation to OTα due to carboxypeptidase activity	[53]
*Bacillus amyloliquefaciens*	ZEN	Adsorption to bacterial cells	[54]
*Bacillus licheniformis*	ZEN	Adsorption to bacterial cells	[55]
*Bacillus megaterium*	OTA	Adsorption to bacterial cells	[56]
*Bacillus pumilus*	ZEN	Biotransformation due to esterase activity	[57]
*Bacillus subtilis*	AFB_1_	Biotransformation into less toxic products due to laccase activity	[58]
*Bacillus subtilis*	DON	NA	[59]
*Bacillus subtilis*	OTA	Adsorption to bacterial cells	[60]
*Bacillus subtilis*	ZEN; 17-β-estradiol	Biotransformation into ZEN-14-phosphate and 17-β-estradiol-3-phosphate	[61]
*Bacillus velezensis*	AFB1	Biotransformation into less cytotoxic products	[62]
*Brevibacterium casei, B. linens, B. iodinum*	OTA	Biodegradation to OTα	[63]
*Cupriavidus numazuensis; C. oxalaticus, C. basilensis, C. metalliduriens*	OTA, AFB1, ZEN, T-2	Biotransformation into undefined products with lower toxicity	[64,65]
*Devosia insulae*	DON	Biotransformation into 3-keto-DON	[66]
*Escherichia coli*	AFB_1_	Biotransformation into less toxic products (C_16_H_14_O_5_ and other metabolites)	[67]
*Enterobacter cloaceae* subsp. *dissolvens*	PAT	Enzymatic biotransformation into E-ascladiol	[68]
*Gluconobacter oxydans*	AFB1, OTA, CIT, PAT	Physical binding to bacterial cell wall proteins and polysaccharides	[69]
*Lactiplantibacillus plantarum*^1^, *Levilactobacillus brevis*^1^, *Fructilactobacillus sanfranciscensis*^1^	OTA	Adsorption into the bacterial cell wall	[70]
*Lactiplantibacillus plantarum* ^1^	PAT	Biotransformation into E-ascladiol	[71]
*Lacticaseibacillus casei* ^1^	PAT	Simultaneous partial biotransformation into an undefined product and adsorption into the bacterial cell wall	[72]
*Lactiplantibacillus plantarum* ^1^	ZEN	NA	[73]
*Lactiplantibacillus plantarum* ^1^	ZEN	Biotransformation due to esterase activity	[74]
*Lysinibacillus* sp.	ZEN	Enzymatic biotransformation	[75]
*Lysobacter* sp.	OTA	Biodegradation to OTα	[76]
*Nocardioides*	DON	Biotransformation into 3-keto-DON and 3-epi-DON as intermediate products	[77]
*Pediococcus parvulus*	OTA	Biodegradation to OTα	[78]
*Pelagibacterium halotolerans*	DON	Biotransformation into less-toxic 3-keto-DON by oxidation of the C3 hydroxyl group	[79]
*Pseudomonas geniculata*	AFB_1_	Non-enzymatic transformation into C_17_H_14_O_7_	[80]
*Rhodococcus pyridinivorans*	AFB_1_	NA	[81]
*Rhodococcus pyridinivorans*	ZEN	Biotransformation into non-estrogenic undefined products	[82]
*Rhodococcus erythropolis, R. rhodochrous, R. pyridinivoran*	AFB_1_, T-2	Biotransformation into undefined non genotoxic products	[83]
*Sphingomonadales* family	FB_1_	Biodegradation into an undefined hydrolyzed product due to carboxyesterase activity	[84]
*Streptomyces* spp.	AFB_1_	Biotransformation into undefined less genotoxic products	[85]
Bacterial consortium isolated from soil (*Methylophilus; Hyphomicrobium; Ancylobacter; Pseudomonas; Prosthecomicrobium; Taonella; Bosea,* and other genera	DON	Biotransformation into 3-keto-DON	[86]
Microorganisms from Kefir grains (*Lentilactobacillus kefiri* ^1^*, Kazachstania servazzii* ^2^ and *Acetobacter syzygii)*	AFB_1_, ZEN, OTA	Adsorption	[87]
Bacterial consortium consists of *Geobacillus, Tepidimicrobium, Clostridium* and *Aeribacillus*	AFB_1_, ZEN,	NA	[88]
Bacterial consortium isolated from spent mushroom compost: *Pseudomonas, Comamonas*, *Delftia, Sphingobacterium*, *Achromobacter*	FB_1_	Enzymatic transformation into low-toxicity metabolites	[89]
Yeasts			
*Candida guilliermondii*	PAT	Biotransformation into E-ascladiol with short-chain dehydrogenase/reductase	[90,91]
*Candida parapsilosis*	ZEN	Biotransformation into less toxic β-zearalenol (β-ZOL) and zearalenone-14,16-diglucosid	[92]
*Candida utilis*	ZEN, OTA, AFB_1_	Adsorption into cell wall preparation	[93]
*Kodameae ohmeri*	PAT	Biotransformation into E- and Z-ascladiol	[94]
*Komagataella phaffi*	FB_1_	Biotransformation due to fumonisin esterase	[95]
*Meyerozyma guilliermondii*	PAT	Biotransformation into undefined products	[96]
*Metschnikowia pulcherrima*	PAT	Biodegradation	[97]
*Metschnikowia pulcherrima*	OTA	Biotransformation into undefined productsOTα and sodium adduct of OTα with loss of the coumarin group	[98,99]
*Pichia caribbica*	PAT	Enzymatic biodegradation	[100]
*Rhodosporidium kratochvilovae*	PAT	Biotransformation into desoxypatulinic acid	[101,102]
*Rhodotorula mucilaginosa*	PAT	Enzymatic biotransformation by orotate phosphoribosyltransferase	[103,104]
*Saccharomyces cerevisiae*	PAT	Adsorption to proteins and polysaccharides in the cell walls	[105]
*Saccharomyces cerevisiae*	OTA	Adsorption by cell wall polysaccharides	[106,107]
*Saccharomyces cerevisiae*	OTA	Adsorption	[108]
*Saccharomyces pastorianus*	DON, HT-2, T-2	Biodegradation and/or adsorption	[109]
*Saccharomyces cerevisiae, S. pastorianus*	DON, ZEN	Adsorption by yeast cells	[110]
*Saccharomyces cerevisiae* thermal inactivated cells powder	AOH, AME	Adsorption	[111]
*Yarrowia lipolytica*	OTA	Biodegradation into less toxic products	[112]
Microorganisms isolated from Kombucha culture: *Pichia occidentalis*, *Candida sorboxylosa* and *Hanseniaspora opuntiae*	AFB_1_	Biodegradation into less toxic products	[113]
Molds			
*Aspergillus niger*	OTA	Biodegradation into ochratoxin α by extracellular ochratoxinase	[114]
*Aspergillus niger*	AFB1	Biodegradation into AFB_2_-GOH	[115]
*Byssochlamys nivea*	PAT	Biodegradation	[116,117]
*Clonostachys rosea*	ZEA	Biotransformation into a less toxic product due to lactonase activity followed by decarboxylation	[118]
*Rhizopus oryzae, Trichoderma reesei*	AFs	Biodegradation	[119]
*Cladosporium uredinicola*	AFB1	Biotransformation into less cytotoxic products	[120]

^1^ formerly belonging to *Lactobacillus* genera. AFB_1_—aflatoxin B_1_; OTA—ochratoxin A; PAT—patulin; ZEN—zearalenone; DON—deoxynivalenol; CIT—citrinin; FB_1_—fumonisin B_1_; AOH—alternariol; AME—alternariol monomethyl ether; AFB_2_-GOH—AFB2 coupling with glutathione; ^2^ yeasts strain; NA—detailed data unavailable.

## Data Availability

Not applicable.
